# Is Ti-Coated PEEK Superior to PEEK for Lumbar and Cervical Fusion Procedures? A Systematic Review and Meta-Analysis

**DOI:** 10.3390/jcm14217696

**Published:** 2025-10-30

**Authors:** Julia Kincaid, Richelle J. Kim, Akash Verma, Ryan W. Turlip, David D. Liu, Daksh Chauhan, Mert Marcel Dagli, Richard J. Chung, Hasan S. Ahmad, Yohannes Ghenbot, Ben Gu, Jang Won Yoon

**Affiliations:** 1 Department of Neurosurgery, Perelman School of Medicine, University of Pennsylvania, Philadelphia, PA 19104, USA; kincaidj@sas.upenn.edu (J.K.); rjk2184@columbia.edu (R.J.K.); akverma@sas.upenn.edu (A.V.); daksh.chauhan@pennmedicine.upenn.edu (D.C.); marcel.dagli@pennmedicine.upenn.edu (M.M.D.); hasan.ahmad@pennmedicine.upenn.edu (H.S.A.); yohannes.ghenbot@pennmedicine.upenn.edu (Y.G.); ben.gu@pennmedicine.upenn.edu (B.G.); 2 Department of Neurosurgery, Mass General Brigham, Harvard Medical School, Boston, MA 02114, USA; ddliu@mgb.org; 3 School of Medicine, University of Virginia Health, Charlottesville, VA 22908, USA; mbm7eu@virginia.edu; 4 Department of Neurosurgery, University of California San Francisco, San Francisco, CA 94143, USA

**Keywords:** cage materials, fusion rates, Polyetheretherketone (PEEK), spinal fusion

## Abstract

**Background/Objectives**: Utilization of polyetheretherketone (PEEK) cages for spinal fusion has surged in the U.S., yet comprehensive comparisons evaluating its postoperative effectiveness with alternative materials remain limited. This systematic review investigates the efficacy of PEEK cages against traditional fusion materials across various surgery types, elucidating PEEK’s impact on fusion rates, postoperative outcomes, and long-term success. **Methods**: A systematic search of PubMed, CINAHL, Scopus, Embase, and Web of Science was conducted through 14 October 2024. Included studies were randomized controlled trials (RCTs) comparing PEEK cages with titanium, silicon nitride, and metal-coated PEEK cages for anterior cervical discectomy and fusion (ACDF), posterior lumbar interbody fusion (PLIF), and transforaminal lumbar interbody fusion (TLIF). Article quality was assessed using GRADE criteria. **Results**: From 288 initially screened articles, 25 RCTs involving 2046 patients (mean follow-up 23.1 ± 18.2 months) met inclusion criteria and were determined as moderate (n = 21) or high (n = 4) quality. Fusion rates by cage material for PEEK (n = 1041), Ti-PEEK (n = 291), and titanium (n = 53) were 85.63 ± 18.00%, 80.05 ± 19.9%, and 92.75 ± 11.31%, respectively. In ACDF, titanium cages achieved higher fusion rates than PEEK (100% vs. 94%). In PLIF and TLIF, coated PEEK outperformed uncoated PEEK (75% vs. 71% and 94% vs. 84%, respectively). Uncoated PEEK achieved fusion rates of 94.04 ± 5.04% for ACDF, 71.21 ± 21.93% for PLIF, and 83.50 ± 24.66% for TLIF, with titanium outperforming PEEK in early fusion outcomes. Coated PEEK demonstrated potential improvements in fusion rates over uncoated PEEK in PLIFs and TLIFs. **Conclusions**: Selection of cage material for spinal fusions should be tailored to surgical requirements and patient needs. While titanium and PEEK are effective, their performance varies across contexts. New materials and surface modifications may enhance these outcomes further, warranting future research in long-term studies and development of novel materials. These findings can help surgeons choose cage materials according to procedure type, patient characteristics, and imaging needs.

## 1. Introduction

Spinal fusion is a widely accepted surgical approach essential for treating pathologies such as scoliosis, degenerative spondylolisthesis, and lumbar and cervical stenosis [1,2,3]. In recent decades, the global incidence of spinal fusion surgeries has risen alongside an aging population. In the United States, the rate of cervical and lumbar fusions has increased by 89% and 134%, respectively, reflecting the large demand for treatment of debilitating spinal pathologies [4,5,6,7].

Currently, anterior cervical discectomy and fusion (ACDF), transforaminal lumbar interbody fusion (TLIF), and posterior lumbar interbody fusion (PLIF) are among the most common techniques for spinal fusion [1,7,8]. Although each procedure involves distinct features, the adoption of interbody cages has revolutionized these approaches, leading to reduced postoperative pain, fewer complications, and increased fusion rates compared to traditional bone grafts [9,10,11,12].

Various materials for interbody cages exist, with polyetheretherketone (PEEK) and titanium alloy being the most frequently used [12,13,14]. In the 1990s, PEEK arose as a commonly used graft due to its unique characteristics which mimic the biomechanical and biological properties of natural bone [14,15,16,17]. PEEK possesses an elastic modulus similar to cortical bone, enabling the reduction of stress-shielding effects. Another advantage of PEEK is its radiolucency which allows for enhanced assessment of fusion imaging [15,16,18,19,20,21].

The literature currently lacks comprehensive, controlled studies that assess the postoperative efficacy of PEEK cages against other fusion materials. Earlier reviews were either not restricted to randomized controlled trials or limited to a single procedure, which reduced the strength and generalizability of their conclusions. While there are reports on specific comparisons, a broader analysis across surgery types is missing. This gap underscores the critical need for detailed comparative research on fusion rates, post-surgical outcomes, and long-term effectiveness of various cage materials. Accordingly, this systematic review, limited exclusively to randomized controlled trials across ACDF, PLIF, and TLIF, aims to compare fusion rates and peri-operative outcomes of PEEK to other commonly used fusion cage materials, thereby guiding cage selection and operative decision-making across surgical approaches.

## 2. Materials and Methods

### 2.1. Information Sources and Search Strategy

This systematic review was designed in accordance with Preferred Reporting Items for Systematic Reviews and Meta-Analyses (PRISMA) guidelines (Supplementary Materials). A search for the variable uses and applications of PEEK for spinal surgery was performed using PubMed, CINAHL, Scopus, Embase, and Web of Science from database inception through 14 October 2024. Our search terms are as follows: (“Cage” OR “Cages”) AND ((“Titanium Alloy (TiAl6V4)” OR “Porous Titanium” OR TiAl6V4) OR “Carbon Fiber Reinforced Polymer” OR (“Polyetheretherketone” OR “PEEK”) OR (3D-printed)) AND (“Fusion” OR “Spine Fusion” OR “Spinal Fusion” OR “Lumbar “OR “Lumbar Fusion” OR “Lumbar Interbody Fusion”).

### 2.2. Inclusion and Exclusion Criteria

For this systematic review, all randomized controlled trials (RCTs) comparing PEEK and other fusion materials such as titanium and metal-coated PEEK cages for ACDF, PLIF, and TLIF were included. The primary outcome of analysis was postoperative fusion rates. The following exclusion criteria were applied:

1. Non-English studies;
2. Non-original articles such as commentaries, systematic reviews, or meta-analysis;
3. Studies lacking fusion rates following surgical intervention;
4. Study designs other than RCTs;
5. Studies lacking PEEK comparison.

### 2.3. Article Selection Process

The software Covidence (Veritas Health Innovation Ltd., Melbourne, Australia) was used for the review organization. Articles were screened by title and abstract followed by full-text analysis applying the inclusion and exclusion criteria. At each step, two independent reviewers (JK, AV) screened all articles. If a consensus could not be reached, a third reviewer (RK) adjudicated, resolving any conflicts and making a final decision to prevent bias in the article selection process. No authors with conflicts of interest participated in data extraction or quality grading.

### 2.4. Data Extraction

The primary outcome measure was postoperative fusion rate, quantified by the proportion of patients that were deemed “fused” by the study. Other recorded data included study demographics (author, year of publication, article type, surgery type, number of patients, patient sex, and average patient age), objective outcomes (blood loss, hospital stay length, operative time, and follow-up time), and subjective outcomes (Visual Analog Scale [VAS] scores and Oswestry Disability Index [ODI] scores). Studies that provided both preoperative and postoperative values for patient-reported outcome measures (PROMs) were included in the analysis. Studies that did not separate VAS reporting into VAS Neck, Arm, Leg, or Back were excluded from the VAS analysis. To ensure consistency, standardized definitions for case and control groups were established and applied uniformly across all included studies. The case group was defined as the uncoated PEEK cage, and the control group was defined as the coated PEEK and non-PEEK cages. Values were reported as frequency-weighted means (FWM). For consistency, we extracted outcomes at the latest reported follow-up time as definitions of early vs. long-term fusion varied across studies.

### 2.5. Meta-Analysis

Random-effects meta-analyses for (i) fusion rate (proportion fusion) and (ii) patient-reported outcomes (VAS and ODI) were performed. For fusion, study-level proportions were pooled using inverse-variance random effects after variance-stabilizing transfomrion; for PROMs, we pooled mean change from baseline (or post-op means when change was unavailable) using random-effects models. Heterogeneity was summarized by I^2^ and τ^2^, and subgroup analyses were prespecified by procedure (ACDF, PLIF, TLIF) and cage material (uncoated PEEK, coated PEEK, titanium, other).

### 2.6. Article Quality Grading

Article quality grading for this systemic review was conducted using the Grading of Recommendations Assessment, Development, and Evaluation (GRADE) criteria [22]. GRADE incorporates considerations of inconsistency, risk of bias, imprecision, and publication bias. The studies began with a high-quality grade and remained high unless there was a risk of bias, inconsistency, imprecision, or publication bias, in which case they were downgraded. This review was not registered in PROSPERO due to different registration standards at the time of data collection, posing a limitation in methodology.

## 3. Results

**Figure 1:**
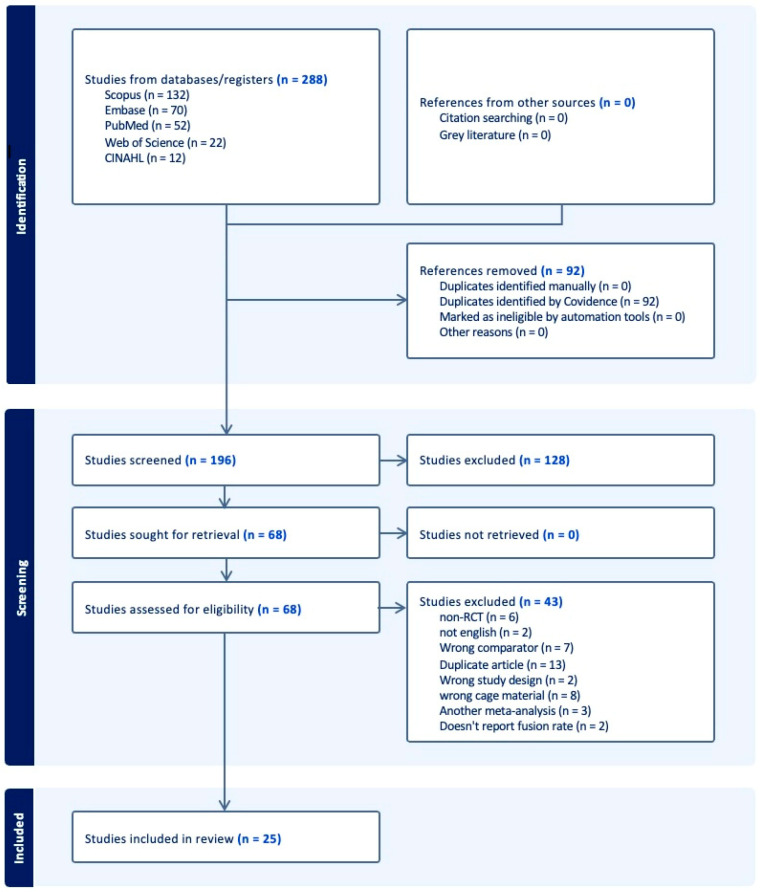
PRISMA diagram.

Our search resulted in 288 articles. After removing duplicates, 196 articles remained. Title and abstract screening narrowed this to 68. After a full-text search, 25 articles met the inclusion criteria and were included in the study, reporting data from 11 countries. All included studies (n = 25) were RCTs and were designated as moderate or high-quality according to the GRADE criteria (Table 1, Figure 1) [23,24,25,26,27,28,29,30,31,32,33,34,35,36,37,38,39,40,41,42,43,44,45,46,47].

### 3.1. Study Demographics

All included patients (n = 2046) had a frequency-weighted mean age of 54.90 ± 7.01 years. Females represented 51.32% of the total cohort. The frequency-weighted postoperative follow-up time for all patients was 23.11 ± 18.15 months. There were three reported categories of surgery types, including ACDF (n = 11), PLIF (n = 6), and TLIF (n = 7) studies for a total of 24 studies. One remaining study compared both ACDF and TLIF. A focused breakdown of procedure characteristics such as blood loss and hospital stay is found in Table 2.

### 3.2. Postoperative Fusion Outcomes in ACDF

**Figure 2:**
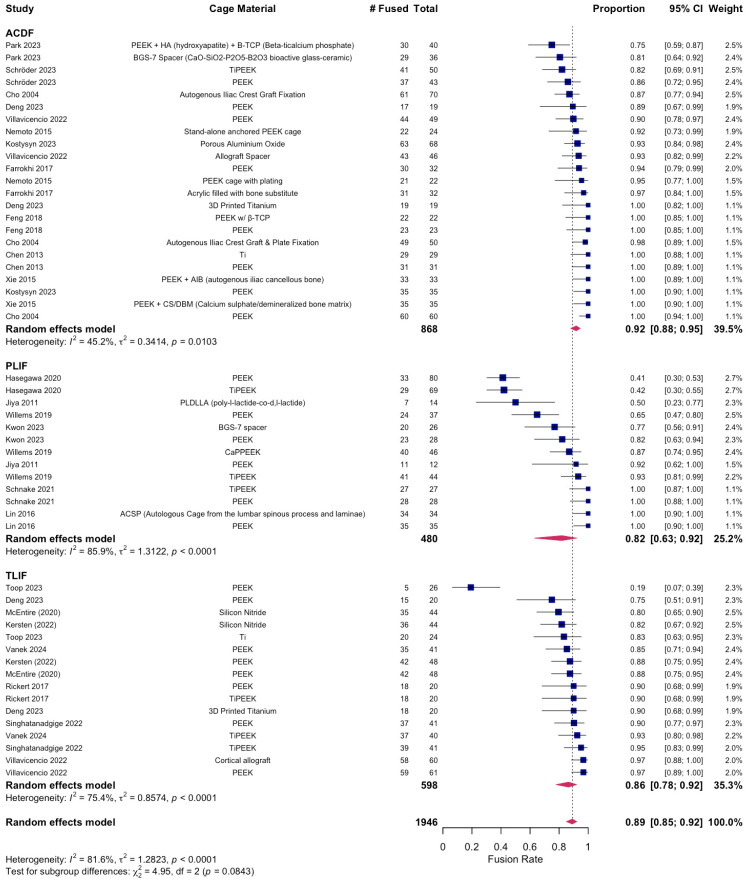
Overall pooled fusion rates stratified by procedure and by material [23,24,25,26,27,28,30,31,32,33,34,35,36,37,38,39,40,41,42,43,44,45,46,47].

**Figure 3:**
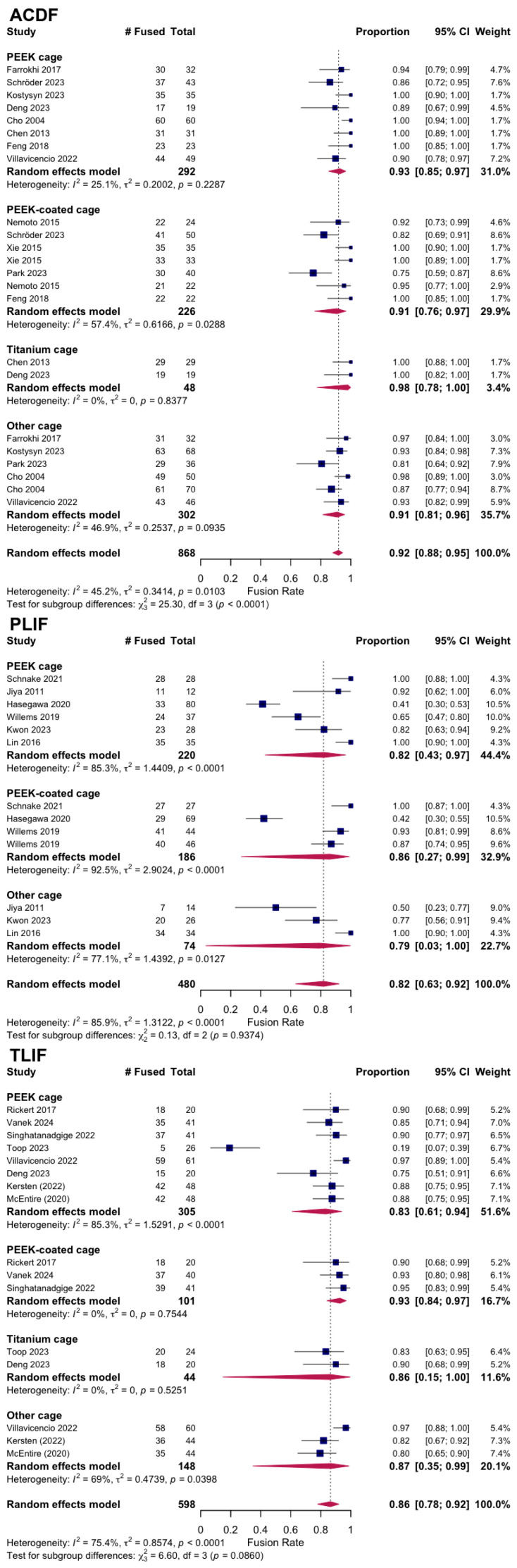
Random-effects meta-analysis of fusion rates by cage material within each procedure (ACDF, PLIF, TLIF) [23,24,25,26,27,28,30,31,32,33,34,35,36,37,38,39,40,41,42,43,44,45,46,47].

**Figure 4:**
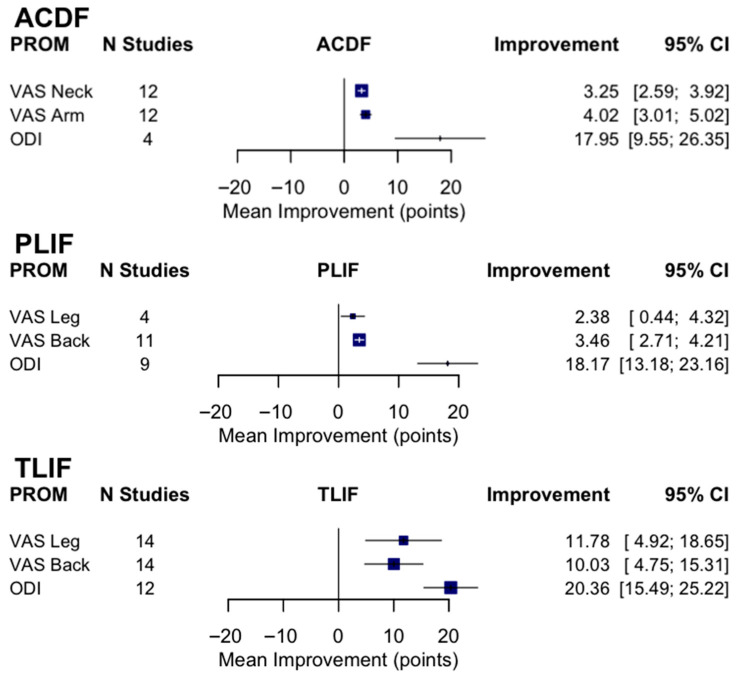
Random-effects pooled mean change in patient-reported outcomes (PROMs) by procedure.

A total of 12 RCTs with a total of 968 patients assessed the efficacy of PEEK against other spinal fusion materials in ACDF procedures. Between uncoated PEEK and titanium cages, Chen et al. found no significant differences in clinical assessment for spinal fusion [25]. Across studies that compared PEEK cages that differed in stabilization properties, coating material, or bone substitutes, no significant differences were observed in fusion rates, subsidence rates, or ODI and VAS Arm/Leg scores between the comparison groups [26,27,32,45]. Notably, 24 months after surgery, the standalone anchored PEEK cage showed lower incidence of adjacent-level ossification than PEEK cages with plating (12.5% vs. 63.6%, *p* = 0.001) [26]. Six studies compared PEEK cages against varying fusion materials in ACDF patients [23,29,30,39,42,44]. There were no statistical differences in clinical outcomes, fusion and recovery rates, and VAS Arm/Neck between PEEK cages vs. porous silicon nitride spacers, allograft spacers, and 3D-printed titanium cages [27,32,33]. Similarly, no differences were observed between PEEK cages filled with a mixture of hydroxyapatite (HA) and β-tricalcium phosphate (β-TCP) and CaO-SiO_2_-P_2_O_5_-B_2_O_3_ glass–ceramic (BGS-7) spacers [23,29,39,41]. In contrast, Kostysyn et al. found that at 18 months, PEEK cages had higher fusion rates (100% vs. 92.6%) than the porous aluminum oxide cages and reported enhanced grade I fusion in the PEEK group over the aluminum oxide group (77.1% vs. 39.7%, *p* < 0.001) at the final follow-up (Table 3). Additionally, the pooled fusion rate was 0.92 (95% CI 0.88–0.95; I^2^ = 45.2%, τ^2^ = 0.34, *p* = 0.01), indicating moderate heterogeneity (Figure 2). Subgroup analyses demonstrated significant differences by cage material (χ^2^ = 25.30, df = 3, *p* < 0.0001). Pooled fusion rates were 0.93 [0.85–0.97] for PEEK, 0.91 [0.76–0.97] for coated PEEK, 0.98 [0.78–1.00] for titanium, and 0.91 [0.81–0.96] for other cage types (Figure 3). All materials performed well, with titanium and uncoated PEEK trending toward the highest estimates. Additionally, pooling across studies, ACDF improved VAS-Neck by +3.25 points [2.59–3.92] and VAS-Arm by +4.02 points [3.01–5.02]; ODI decreased by −17.95 points [−26.35 to −9.55] (Figure 4). Disability reduction was substantial, though ODI estimates are based on fewer studies.

### 3.3. Postoperative Fusion Outcomes in PLIF

A total of 480 patients were analyzed across six RCTs that assessed PEEK’s recovery trends against other fusion materials in PLIF procedures. Three studies compared the outcomes of uncoated PEEK cages with those of TiPEEK and CaP-PEEK cages and did not find significant differences in improvements in ODI and VAS Leg/Back following surgery [33,34,36]. However, Hasegawa et al. found that the bone fusion rates were significantly higher at 6 months after surgery in the TiPEEK group than in the PEEK group in the unadjusted modified intention-to-treat analysis (*p* = 0.03). Willems et al. found that nanocoated PEEK cages had significantly increased fusion rates as compared to PEEK alone after one year (PEEK: 65.6% vs. TiPEEK: 93.9%, *p* = 0.0034, CaP-PEEK: 88.0%, *p* = 0.032). Similarly, at 24 months there were no differences in fusion rates between PEEK and autologous cages using the lumbar spinous process and laminae (ACSP) [28]. However, the subsidence around the BGS-7 cages was significantly less than PEEK (*p* < 0.05) (Figure 3) [43]. The pooled fusion rate was 0.82 (95% CI 0.63–0.92; I^2^ = 85.9%, τ^2^ = 1.31, *p* < 0.0001), reflecting substantial heterogeneity (Figure 2). Subgroup analyses by cage material yielded wide confidence intervals due to the limited number of studies: 0.82 [0.43–0.97] for PEEK, 0.86 [0.27–0.99] for coated PEEK, and 0.79 [0.03–1.00] for other cage types (Figure 3). Because of heterogeneity and the wide confidence intervals, no clear differences could be determined between cage types in PLIF. In PLIF, VAS-Leg improved by +2.38 points [0.44–4.32] and VAS-Back by +3.46 points [2.71–4.21]. ODI decreased by −18.17 points [−23.16 to −13.18] (Figure 4).

### 3.4. Postoperative Fusion Outcomes in TLIF

A total of 598 patients were analyzed across eight RCTs that assessed PEEK against other fusion materials in TLIF procedures (Table 3, Figure 2). Toop Et Al. compared PEEK cages with activated titanium, a form of titanium cage that has received micro- and nanoscale internal and topographic cage modifications. In contrast to PEEK at 6 months, titanium cages optimized postoperative fusion (84.0% vs. 20.6%, *p* < 0.001) and had lower rates of subsidence (20.8% vs. 41.4%, *p* < 0.001) [46]. Three studies explored the clinical effects of utilizing titanium-coated PEEK (TiPEEK) over uncoated PEEK [31,38,47]. Out of the three studies, Vanek et al. found subsidence rates higher in the PEEK group (26.8%) than the TiPEEK group (5%) (*p* = 0.007), and Singhatanadgige et al. noted that the TiPEEK cohort demonstrated a higher fusion rate than PEEK at 6 months postoperation (91.8% vs. 76%; *p* = 0.03), with no difference in fusion rate and cage subsidence at 12 months. Two studies comparing Si_3_N_4_ with PEEK as the fusion cage material found no significant difference in fusion rates and VAS Leg/Back [35,37]. Villavicencio et al. reported comparable fusion rates for PEEK interbody lordotic spacers to cortical allograft spacers at 24 months (97.5% vs. 97.5%), and while all other clinical outcomes for pain and radiographic outcome data were equally positive with significant improvements in all measured outcomes (*p* < 0.0001), no differences were detected between the groups at any of the follow-up times [40]. Deng et al. observed statistically similar fusion rates between 3D-printed titanium cages (3DPT) and PEEK at 6 months (92.3% vs. 75%, *p* = 0.225) but superior bone–cage interface contact (15.4% vs. 75%, *p* < 0.001) and lower subsidence (0.7 ± 0.3 mm vs. 1.5 ± 0.8 mm, *p* < 0.001) for the 3DPT (Table 4, Figure 3) [41]. The pooled fusion rate was 0.86 (95% CI 0.78–0.92; I^2^ = 75.4%, τ^2^ = 0.86, *p* < 0.0001) (Figure 2). Subgroup analyses by cage material showed pooled fusion of 0.83 [0.61–0.94] for PEEK, 0.93 [0.84–0.97] for coated PEEK, 0.86 [0.15–1.00] for titanium, and 0.87 [0.35–0.99] for other cage types (Figure 3). Although confidence intervals overlapped, coated PEEK tended to outperform uncoated PEEK in TLIF. TLIF demonstrated greater absolute improvements than the other procedures, with VAS-Leg improving by +11.78 points [4.92–18.65], VAS-Back by +10.03 points [4.75–15.31], and ODI decreasing by −20.36 [−25.22 to −15.49] (Figure 4).

**Figure 5:**
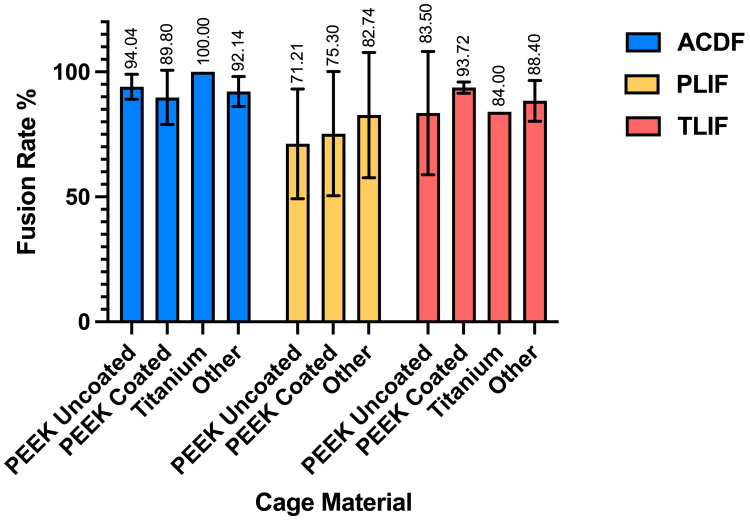
Bar graph of fusion rates by surgery type and fusion cage material.

**Figure 6:**
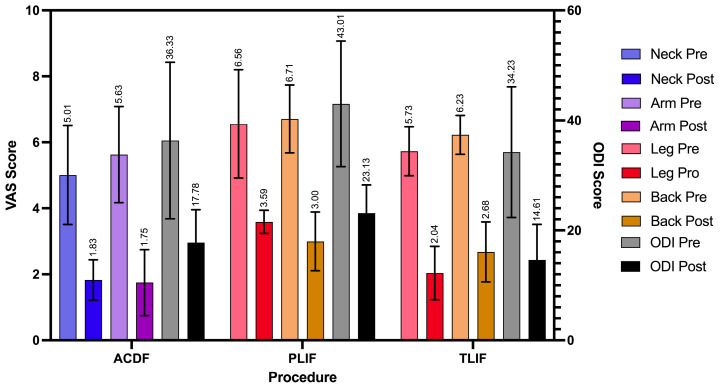
Bar graph of VAS (Neck, Arm, Leg, Back) and ODI scores pre- and postoperation. Studies assessing ACDF typically reported ODI scores. However, three reported Neck Disability Index (NDI) and were included in the analysis.

Taken together, we found high fusion rates overall but with differences by procedure and cage type: across 1946 patients, the pooled fusion rate was 89% [95% CI: 85–92%], though heterogeneity was substantial (I^2^ = 81.6%) (Figure 5). These analyses demonstrate consistently high fusion rates after ACDF, more variable rates after PLIF and TLIF, and procedure-specific trends suggesting that cage material may influence outcomes (Figure 2, Figure 3, Figure 4, Figure 5 and Figure 6).

## 4. Discussion

### 4.1. Study Trends

This systematic review compares the postoperative fusion rates and perioperative outcomes of PEEK with other fusion materials after ACDF, TLIF, and PLIF. After reviewing 25 RCTs, we observed varying outcomes regarding the efficacy of PEEK, coated PEEK, titanium, and other materials across procedures. For ACDF, 12 included studies often demonstrated high fusion rates for titanium, with all included subgroups of the material achieving 100% fusion. While some patient groups treated with PEEK or coated PEEK achieved 100% fusion, the average fusion rates were slightly below 100%. Uncoated PEEK performed slightly better than coated PEEK in ACDF procedures. Contrarily, the results from the six included PLIF studies show that coated PEEK outperforms uncoated PEEK in achieving higher fusion rates. Similarly, in the included studies for TLIF, coated PEEK reported higher fusion rates compared to uncoated PEEK, with titanium reporting fusion rates between the two. However, this was not uniformly the case, as some studies for both PLIF and TLIF reported no significant differences between titanium-coated and uncoated PEEK. Additionally, the heterogeneity of included trials and small sample sizes serves as a limitation for making definitive conclusions. This review demonstrates that the performance of PEEK, coated PEEK, and titanium is context-dependent, and there is no clear difference in fusion rates between the titanium and titanium-coated PEEK interbodies for achieving successful fusion. These results also highlight the advantages of material and design-specific modifications to titanium and PEEK cages in PLIFs and TLIFs.

### 4.2. Historical Trends

The historical evolution of spinal fusion materials provides critical context to this review’s findings. When spinal fusions via interbody cages first became popularized, titanium was among the earliest materials used due to its strength, corrosion resistance, biocompatibility, and bone ingrowth promotion [18,48,49,50]. However, titanium possesses a mismatch in the elastic modulus of the vertebrae and cortical bone, which can lead to stress shielding and subsequent graft subsidence [18,48,49,50]. Additionally, the high radiodensity of titanium causes imaging artifacts that make postoperative imaging difficult to assess fusion status [18,50]. These challenges align with this review’s findings, where titanium showed strong early fusion outcomes but was occasionally associated with higher subsidence rates compared to bioactive-coated PEEK.

In response to limitations of titanium interbody cages, PEEK emerged in the early 1990s in the hopes of increasing fusion rates [49]. Unlike titanium, PEEK has a modulus of elasticity similar to vertebrae and cortical bone, causing less stress shielding and subsidence [18,48,49,50]. Additionally, PEEK displays no radiographical artifacts, contributing to greater accuracy with postoperative imaging [18]. Due to these benefits, PEEK has become the most commonly used interbody material since the late 1990s [49]. Evidenced in this review, this trend is for good reason: At 18 months, the uncoated PEEK cage group had higher fusion rates and grade I fusion than the porous aluminum oxide cage group in ACDF [42]. Additionally, the majority of ACDF studies reported no significant differences in clinical assessment between PEEK and other cage materials. In two studies, PEEK also demonstrated lower complication rates or incidence of adjacent-level ossification, proving the reliability of PEEK cages. However, because PEEK is bioinert and hydrophobic, it has a limited osteointegration capacity which impedes fusion capability [18,48,49,50]. Additionally, potential biofilm formation of PEEK cage surfaces can disrupt binding to the host bone which can impede solid fusion [48]. Thus, continued innovation has introduced coated PEEK cages to preserve the modulus of elasticity of PEEK while promoting bioactivity.

Combining the benefits of titanium and PEEK interbody cages, TiPEEK has become a popular material for spinal fusions. TiPEEK, a version of titanium-coated PEEK, was created to increase the osteointegration of uncoated PEEK, one of PEEK’s drawbacks [51,52]. Other bioactive materials, such as hydroxyapatite and calcium, have also been used to coat PEEK in hopes of promoting its bioactivity [33,53].

Likewise, this study also analyzes postoperative outcomes for PEEK and coated PEEK, allowing for comparisons in fusion rates and clinical outcomes. While PEEK shows clinically acceptable fusion rates for ACDF, PLIF, and TLIF, its ability to be modified by coatings allows for enhanced bioactivity which can lead to greater fusion rates compared to titanium and uncoated PEEK. These significant improvements in fusion outcomes observed with studies’ modifications to PEEK, such as TiPEEK and CaP-PEEK, suggest closed performance gaps specifically in PLIF and TLIF, supporting their clinical relevance. Amidst these advancements, the landscape of spinal fusion materials has continued to diversify. Materials such as bioactive ceramics, 3D-printed titanium, and acrylic cages are being explored for their potential to improve fusion rates and biocompatibility [18,29,30]. In this systematic review, advancements in titanium, such as activated and 3D-printed titanium cages, demonstrate advantages in fusion rates, subsidence, and bone–cage interface contact compared to traditional PEEK cages in TLIFs. In addition, bioactive ceramics such as silicon nitride demonstrated comparable fusion outcomes to PEEK with potential advantages in reducing imaging artifacts. These developments underscore a broader trend in biomedical engineering towards materials that can actively contribute to biological processes, potentially leading to faster healing and better long-term outcomes.

### 4.3. Upcoming Materials

Although progress has been made with spinal fusion materials, challenges remain in achieving optimal fusion, particularly in complex cases or in patients with underlying health. As the field progresses, new materials are being explored in the hopes of improving long-term outcomes, including improvements in 3D printing. Recent technical improvements have created the opportunity for 3D printing with PEEK, in which a nano-rough surface with antibacterial characteristics can promote fusion similarly to TiPEEK [18]. Another future direction includes bioactive glass which aims to provide a balance of strength, flexibility, and bioactivity that could potentially outperform current materials [44,54]. Additionally, more recent studies have shown that 3D-printed tantalum might be a future direction for cage materials, as early results show that tantalum promotes osseointegration and excellent intervertebral fusion [55]. The integration of biologically active agents directly into the cage materials, such as TiPEEK and CaP-PEEK, also possesses the potential for future improvement in fusion surgery outcomes.

### 4.4. Clinical Implications and Decision-Making Framework

Given the comparable fusion rates and clinical outcomes associated with PEEK, coated PEEK, and titanium cages, material selection should be guided by procedure type, patient characteristics, and imaging needs. For ACDF procedures, uncoated PEEK may be beneficial due to its radiolucency and reduced imaging artifact, therefore facilitating postoperative assessment of fusion status. Conversely, coated PEEK and titanium cages may offer improved early fusion rates in PLIF and TLIF procedures and may be advantageous in surgical cases where enhanced osteointegration is desired. Furthermore, surgeon familiarity, cost factors, and institutional availability remain key aspects for consideration in cage selection. Recent studies have shown that overall hospital costs for spinal fusion performed with PEEK cages have been slightly lower than those of titanium cages, and cost may be a deciding factor for some patients and surgeons [56]. Additionally, some studies have shown that titanium coatings may shear off coated PEEK cages, posing a possible risk to patients, which might also impact surgical decisions of which interbody cages to use [57]. This individualized, context-dependent approach may enhance surgical planning and optimize patient outcomes across spinal fusion procedures. However, with continuous improvements to PEEK technology, PEEK possesses the ability to become an even more effective material for spinal fusion surgeries, possibly overtaking other cage materials.

### 4.5. Limitations

Although our study synthesizes findings from multiple RCTs assessing the efficacy of various spinal fusion materials, there are limitations that should be acknowledged. The selected RCTs contain variance in patient populations, surgical techniques, and follow-up duration, complicating the generalizability of the results. Outcome measures like blood loss and operative time are also heavily influenced by the complexity of the surgical case, introducing additional variability to the RCTs. Additionally, some studies lack detailed demographic data which limits our understanding of results across various populations, thus limiting our ability to create patient-specific treatment strategies. Furthermore, many trials have relatively small sample sizes and most RCTs have follow-up periods shorter than 24 months, limiting our ability to draw definitive conclusions about the long-term effectiveness and safety of the materials studied. Additionally, we were unable to stratify by early vs. long-term follow-up because studies used inconsistent timepoints and definitions of fusion; instead, we prioritized each trial’s final reported follow-up to maximize comparability. Heterogeneity is high in lumbar procedures (PLIF AND TLIF), which reflects variability in study design, patient populations, and outcome reporting. Furthermore, the assessment methods for bone fusion vary among the included studies, adding to the heterogeneity in these studies. As such, while our pooled estimates provide an overall synthesis, they should be considered in the context of underlying heterogeneity across studies. In addition, not all included trials reported measures of variance or subgroup sample sizes for key perioperative outcomes (e.g., operative time, hospital stay, blood loss in PLIF). Lastly, some studies might be subject to publication bias, as studies with positive outcomes are more likely to be published, skewing the data towards more favorable results.

## 5. Conclusions

The findings of this systematic review illustrate the postoperative efficacy of PEEK cages and other commonly used fusion materials across a wide array of surgical approaches. The analysis of 25 RCT studies revealed that both uncoated and coated PEEK and titanium show clinically acceptable fusion rates in specific scenarios, but their efficacy can vary depending on the surgical context and material properties. While titanium cages demonstrate the highest fusion rates for ACDF procedures, there is no clear difference in fusion rates between titanium and coated PEEK for both TLIF and PLIF procedures, additionally confirmed by meta-analytic pooling. This study demonstrates that PEEK has acceptable baseline fusion rates for all procedures, and the introduction of coatings to PEEK can further increase fusion rates, principally in PLIF and TLIF. Thus, as improvements are made to PEEK technology, PEEK possesses the ability to become an even more effective material for spinal fusion surgeries, overtaking previously used cage materials. However, given the variability observed across different types of procedures and materials, it is imperative that future research continues to explore and define the optimal conditions under which each material can achieve the best outcomes. More detailed studies are required to substantiate these findings and help refine surgical practices, ultimately enhancing patient recovery and long-term success in spinal fusion surgeries.

## Figures and Tables

**Table 1:** Baseline characteristics of included studies.

Author(s), Year	Procedure	Control Group	Control (N)	Case Group	Case (N)	Country	Final Quality Grade
Cho et al., 2004 [23]	ACDF	(1) AICG fixation (2) AICG and plate fixation	(1) 70 (2) 50	PEEK cage	60	China	Moderate
Jiya et al., 2011 [24]	PLIF	PLDLLA cage	14	PEEK cage	12	The Netherlands	Moderate
Chen et al., 2013 [25]	ACDF	Titanium cage	29	PEEK cage	31	China	High
Nemoto et al., 2015 [26]	ACDF	PEEK cage with plating	22	Standalone anchored PEEK cage	24	Japan	Moderate
Xie et al., 2015 [27]	ACDF	PEEK cage containing IAB	33	PEEK cage containing CS/DBM	35	China	High
Lin et al., 2016 [28]	PLIF	ACSP	34	PEEK cage	35	China	Moderate
Arts et al., 2017 [29]	ACDF	Porous silicon nitride spacer	52	PEEK cage containing autograft from osteophyte	48	The Netherlands	Moderate
Farrokhi et al., 2017 [30]	ACDF	Acrylic cage filled with bone substitute	32	PEEK cage	32	Iran	Moderate
Rickert et al., 2017 [31]	TLIF	TiPEEK cage	20	PEEK cage	20	Germany	Moderate
Feng et al., 2018 [32]	ACDF	PEEK cage containing β-TCP	22	PEEK cage	23	Taiwan	Moderate
Willems et al., 2019 [33]	PLIF	(1) TiPEEK cage (2) CaP-PEEK cage	(1) 44 (2) 46	PEEK cage	37	Belgium	Moderate
Hasegawa et al., 2020 [34]	PLIF	TiPEEK cage	69	PEEK cage	80	Japan	Moderate
McEntire et al., 2020 [35]	TLIF	Silicon nitride cage	44	PEEK cage	48	USA	Moderate
Schnake et al., 2021 [36]	PLIF	TiPEEK cage	27	PEEK cage	28	Germany	Moderate
Kersten et al., 2014 [37]	TLIF	Silicon nitride cage	44	PEEK cage	48	The Netherlands	Moderate
Singhatanadgige et al., 2022 [38]	TLIF	TiPEEK cage	41	PEEK cage	41	Thailand	Moderate
Villavicencio et al., 2022 [39]	TLIF	Cortical allograft	60	PEEK cage	61	USA	High
Villavicencio et al., 2022 [40]	ACDF	Cortical allograft spacer	46	PEEK lordotic spacer	49	USA	Moderate
Deng et al., 2023 [41]	(1) ACDF (2) TLIF	3DPT cage	(1) 19 (2) 20	PEEK cage	(1) 19 (2) 20	China	Moderate
Kostysyn et al., 2023 [42]	ACDF	Porous aluminum oxide cage	68	PEEK cage	35	Czech Republic	Moderate
Kwon et al., 2023 [43]	PLIF	BGS-7 spacer	26	PEEK cage	28	South Korea	Moderate
Park et al., 2023 [44]	ACDF	BGS-7 spacer	36	PEEK cage containing HA and β-TCP	40	South Korea	Moderate
Schröder et al., 2023 [45]	ACDF	TiPEEK cage	50	PEEK cage	43	Germany	Moderate
Toop et al., 2023 [46]	TLIF	Titanium cage	24	PEEK cage	26	USA	Moderate
Vanek et al., 2024 [47]	TLIF	TiPEEK cage	40	PEEK cage	41	Czech Republic	High

Abbreviations: ACDF, anterior cervical discectomy and fusion; PLIF, posterior lumbar interbody fusion; TLIF, transforaminal lumbar interbody fusion; PEEK, polyetheretherketone; TiPEEK, pitanium-coated polyetheretherketone; BGS-7, CaO-SiO_2_-P_2_O_5_-B_2_O_3_ glass–ceramic spacer; ACSP, Autologous Cage using Spinous Process/Laminae.

**Table 2:** Focused table of patient demographics and objective outcomes segmented by procedure type.

Surgery Type	Patients (N)	Males	Females	FWM Age (Years) (N = 25)	FWM Follow-Up Time (Months) (N = 25)	FWM Operative Time (Minutes) (N = 12)	FWM Hospital Stay (Days) (N = 9)	FWM Blood Loss (mL) (N = 12)	FWM Postop Fusion Rate (%) (N = 25)
ACDF	968	530	438	51.64 ± 5.68	30.08 ± 24.01	155.05 ± 99.70	3.17 ± 2.97	85.91 ± 34.42	92.60 ± 6.88
PLIF	480	228	252	56.88 ± 8.62	14.38 ± 5.76	—	—	—	73.55 ± 22.45
TLIF	598	238	360	58.58 ± 5.00	18.84 ± 8.05	178.28 ± 38.08	13.46 ± 17.15	291.78 ± 129.96	86.62 ± 18.16

Abbreviations: FWM, frequency-weighted mean; postop, postoperative.

**Table 3:** Frequency-weighted mean data values for different cage materials for ACDF, PLIF, and TLIF.

		Patients (N)	Age (Years)	Follow-Up (Months)	Blood Loss (mL)	Operative Time (Minutes)	Hospital Stay (Days)	Fusion Rate (%)
ACDF								
	PEEK Uncoated	364	51.25 +/− 6.90	24.62 +/− 27.89	70.47 +/− 27.94	152.95 +/− 109.091	2.25 +/− 3.36	94.04 +/− 5.04
	PEEK Coated	202	51.53 +/− 6.15	21.62 +/− 4.90	77.37 +/− 26.41	79.32 +/− 26.66	6.74 +/− 0.35	89.80 +/− 10.83
	Titanium	29	45.7 +/− 7.2	97.2 +/− 0	—	—	—	100 +/− 0
	Other (3D-Printed, Acrylic, Porous Aluminum, BGS-7, Autogenous Iliac, Allograft Spacer, Porous Silicon Nitride)	373	52.1 +/− 3.93	27.52 +/− 16.04	102.52 +/− 46.28	188.91 +/− 107.78	2 +/− 3.03	92.14 +/− 5.99
PLIF								
	PEEK Uncoated	220	58.11 +/− 10.24	14.56 +/− 6.20	—	—	—	71.21 +/− 21.93
	PEEK Coated	186	56.60 +/− 8.56	12 +/− 0	—	—	—	75.30 +/− 24.82
	Other (Autologous, PLDLLA, BGS-7)	74	53.91 +/− 8.55	19.78 +/− 6.93	—	—	—	82.74 +/− 25.05
TLIF								
	PEEK Uncoated	305	59.35 +/− 5.87	18.89 +/− 8.33	260.56 +/− 73.00	171.38 +/− 59.45	13.89 +/− 18.58	83.50 +/− 24.66
	PEEK Coated	101	63.71 +/− 0	16.75 +/− 6.93	—	—	—	93.72 +/− 2.23
	Titanium	24	61 +/− 0	6 +/− 0	—	227.9 +/− 0	3.9 +/− 0	84 +/− 0
	Other (Silicon Nitride, 3D-Printed, Cortical Allograft)	168	56.99 +/− 2.24	21.86 +/− 9.0	341.37 +/− 150.85	169.5 +/− 31.56	14.30 +/− 19.77	88.40 +/− 8.15

Abbreviations: PEEK, polyetheretherketone; BGS-7, CaO-SiO_2_-P_2_O_5_-B_2_O_3_ glass–ceramic spacer.

**Table 4:** Focused table of frequency-weighted mean reported outcomes segmented by procedure type.

Surgery Type	VAS Neck Pre/Postop (N = 6)	VAS Arm Pre/Postop (N = 6)	VAS Leg Pre/Postop (N = 9)	VAS Back Pre/Postop (N = 12)	ODI Preop (N = 12)	ODI Postop (N = 12)
ACDF	5.01 ± 1.50, 1.83 ± 0.61	5.63 ± 1.46, 1.75 ± 1.00	—	—	36.33 ± 14.23	17.78 ± 5.96
PLIF	—	—	6.56 ± 1.64, 3.59 ± 0.35	6.71 ± 1.03, 3.00 ± 0.89	43.01 ± 11.42	23.13 ± 5.15
TLIF	—	—	5.73 ± 0.74, 2.04 ± 0.81	6.23 ± 0.59, 2.68 ± 0.91	34.23 ± 11.88	14.61 ± 6.48

Abbreviations: VAS, Visual Analog Scale; ODI, Oswestry Disability Index; preop, preoperative; postop, postoperative.

## Data Availability

The original data presented in the study are openly available in PubMed, CINAHL, Scopus, Embase, and Web of Science.

## Supplementary Materials

The following supporting information can be downloaded at: https://www.mdpi.com/article/10.3390/jcm14217696/s1, PRISMA 2020 Checklist [58].

## Abbreviations

The following abbreviations are used in this manuscript:

ACDF	Anterior Cervical Discectomy and Fusion
PLIF	Posterior Lumbar Interbody Fusion
TLIF	Transforaminal Lumbar Interbody Fusion
PEEK	Polyetheretherketone
TiPEEK	Titanium-coated Polyetheretherketone
HA	Hydroxyapatite
β-TCP	Beta-Tricalcium Phosphate
BGS-7	CaO-SiO_2_-P_2_O_5_-B_2_O_3_ Glass-Ceramic Spacer
ODI	Oswestry Disability Index
VAS	Visual Analog Scale
RCT	Randomized Controlled Trial
GRADE	Grading of Recommendations, Assessment, Development, and Evaluation
3DPT	3D-Printed Titanium
ACSP	Autologous Cage using Spinous Process/Laminae
Si_3_N_4_	Silicon Nitride
